# Multi-marker Solid Tumor Panels Using Next-generation Sequencing to Direct Molecularly Targeted Therapies

**DOI:** 10.1371/currents.eogt.aa5415d435fc886145bd7137a280a971

**Published:** 2014-05-27

**Authors:** Michael Marrone, Kelly K Filipski, Elizabeth M Gillanders, Sheri D Schully, Andrew N Freedman

**Affiliations:** Epidemiology and Genomics Research Program, Division of Cancer Control and Population Sciences, National Cancer Institute, NIH, Rockville, Maryland, USA; Epidemiology and Genomics Research Program, Division of Cancer Control and Population Sciences, National Cancer Institute, NIH, Rockville, Maryland, USA; Epidemiology and Genomics Research Program, Division of Cancer Control and Population Sciences, National Cancer Institute, NIH, Rockville, Maryland, USA; Epidemiology and Genomics Research Program, Division of Cancer Control and Population Sciences, National Cancer Institute, NIH, Rockville, Maryland, USA; Epidemiology and Genomics Research Program, Division of Cancer Control and Population Sciences, National Cancer Institute, NIH, Rockville, Maryland, USA

## Abstract

In contemporary oncology practices there is an increasing emphasis on concurrent evaluation of multiple genomic alterations within the biological pathways driving tumorigenesis. At the foundation of this paradigm shift are several commercially available tumor panels using next-generation sequencing to develop a more complete molecular blueprint of the tumor. Ideally, these would be used to identify clinically actionable variants that can be matched with available molecularly targeted therapy, regardless of the tumor site or histology. Currently, there is little information available on the post-analytic processes unique to next-generation sequencing platforms used by the companies offering these tests. Additionally, evidence of clinical validity showing an association between the genetic markers curated in these tests with treatment response to approved molecularly targeted therapies is lacking across all solid-tumor types. To date, there is no published data of improved outcomes when using the commercially available tests to guide treatment decisions. The uniqueness of these tests from other genomic applications used to guide clinical treatment decisions lie in the sequencing platforms used to generate large amounts of genomic data, which have their own related issues regarding analytic and clinical validity, necessary precursors to the evaluation of clinical utility. The generation and interpretation of these data will require new evidentiary standards for establishing not only clinical utility, but also analytical and clinical validity for this emerging paradigm in oncology practice.

## Clinical scenario

Traditional pharmacogenomic applications used to direct molecularly targeted therapy rely on testing tumor tissue for a single genomic marker followed by using tumor-marker specific therapy. There are several established pharmacogenomic applications that are used clinically to aid in treatment decisions for breast, colon, lung and other solid-tumor cancers (Table 1). With advances in high-throughput –omic technologies and plummeting costs of next-generation sequencing (NGS), researchers have begun to move beyond testing single genes, to multi-gene panels, to sequencing the entire human cancer genome in order to better understand the underlying molecular pathways driving tumorigenesis^1^. Cumulative efforts drawing on resources such as The Cancer Genome Atlas (TCGA) have allowed researchers to develop molecular blueprints common across a wide number of cancer types^2^ and have identified multiple genomic alterations or ‘driver-mutations’ linked to biological pathways in cell proliferation, apoptosis, tumor metabolism, and chromatin biology. Current clinical oncology practice, which has emphasized tumor site and histology, is undergoing a paradigm shift towards what some have referred to as “genomics-driven oncology” focusing on these mechanistic pathways^3^.

**Figure:**
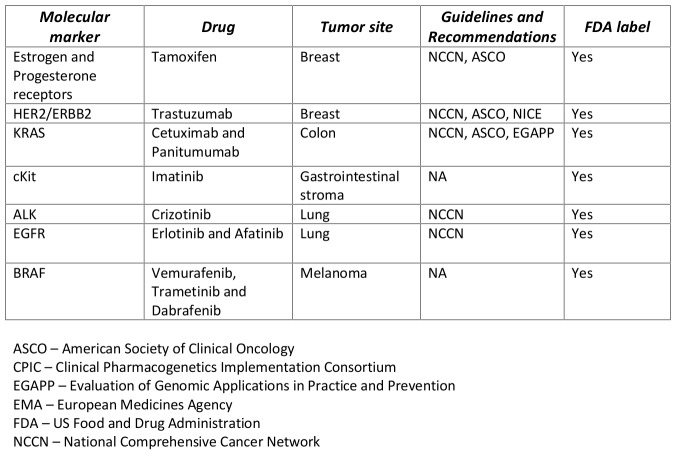
**Table 1. Examples of single-marker single-drug pharmacogenetic associations used in solid-tumor oncology.**

Clinically available NGS tests are used to characterize an individual’s cancer genome through targeted sequencing of pre-specified candidate genes believed to provide clinically actionable molecular targets. Using a single test to detect a broad spectrum of genomic alterations in the biological pathways from a single biopsy is thought to be a more efficient treatment decision process than the single-marker single-treatment approach^4^. The genomics-driven oncology approach using multi-marker panels is intended to expand clinician’s armamentarium to treat patients who may have exhausted standard therapies, especially those with metastatic disease. One key assumption underlying this approach is that a molecular target predictive of treatment response with a currently available therapy in a specific tumor type will have the same clinical effect (predictive of treatment response) in an entirely different tumor type harboring such molecular profile. Complicating this assumption is the reality that additional mutations downstream from the primary molecular target have unknown clinical significance, which may influence treatment response differentially across cancer types. Additional complications arise from molecular heterogeneity within primary tumors as well as secondary tumors^5^, which could lead to limited effectiveness when matching therapies to specific genomic alterations based on a single tumor biopsy.

## Test description

An established clinical test integrating NGS technology for tumor DNA sequencing requires a standardized protocol with details describing pre-analytic, analytic and post-analytic processes. The pre-analytic variables include the patient’s clinical characteristics as well as details describing the collection and preparation of tumor samples. The analytic variables that may affect the precision and accuracy of the targeted sequencing of pre-specified molecular targets (whole-genome, exome, SNPs, etc.) refer to the actual sequencing process itself and are related to the specific NGS platform used to conduct the massively parallel sequencing as well as individual laboratory procedures. The post-analytic variables (i.e. data entry, result validation, interpretation of results, transfer of data and reporting of test results) relevant to NGS include variant calling, functional and clinical interpretation, reporting results to clinicians, and data storage^1^^,^ 6 ^,^ 7. The post-analytic process at the foundation of successfully integrating NGS into clinical practice is variant calling which includes the alignment of the raw tumor sequence data using a reference human genome followed by variant identification from the aligned tumor sequence and molecular annotation of the identified genomic variants with corresponding clinical interpretations. Each of these processes have specialized computational algorithms designed to handle large amounts of sequencing data and to date were primarily developed for research purposes. For clinical applications, such algorithms will need to be adapted to better integrate with the downstream interpretive processes, including clinical decision making^7^.

The expanded reach of NGS platforms provides researchers with a much more comprehensive picture of a tumors genomic architecture. In an effort to leverage the technical advances in sequencing technology and expanded vision of a tumors molecular signature several NGS platforms have been integrated into commercially available solid-tumor sequencing panels to identify clinically actionable variants (Table 2). Despite the lack of consensus as to what constitutes a clinically actionable variant, three general categories may be used to classify variants: 1) variants linked to an FDA-approved drug within a specific tumor type; 2) variants linked to an FDA-approved drug outside of the patients specific tumor type; 3) variants linked to non-FDA approved drugs in preclinical testing or early phase clinical trials^8^. It should be noted variants of unknown significance may also be identified which may not be considered clinically actionable owing to diverse interpretations of what these variants may represent. Similar tests employing NGS technology for directing treatment decisions are available at both academic and community-based cancer treatment centers. The limited commercial availability of the academic and community-based tests fall outside the scope of this review and are not listed in Table 2. As companies refine their workflows and emerging evidence identifies variants common in non-solid tumor, additional liquid-tumor sequencing panels have recently become available, but are not reviewed here.

**Figure:**
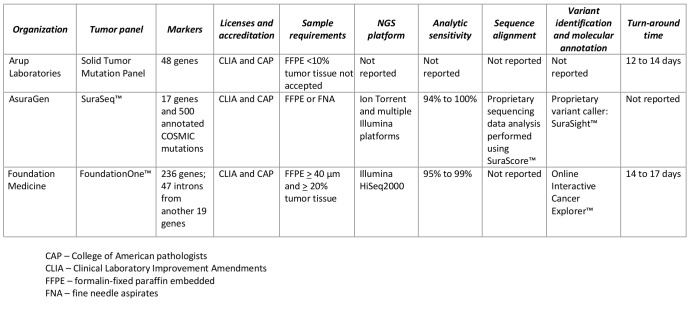
**Table 2. Commercially available multi-marker solid tumor panels using next-generation sequencing (NGS).**

## Public Health importance

To date, the application of multi-marker tumor panels using NGS has primarily focused on individuals with advanced metastatic disease who have exhausted standard therapies for their particular condition. In such cases, these tests may provide unconventional therapeutic options against an otherwise refractory disease. It is unclear at what point this testing and treatment strategy will become a part of the standard molecular profiling of newly diagnosed malignancies, expanding the potential population impact. As evidence emerges on driver-mutations common across a number of cancer types, newly developed molecularly targeted therapies will provide a means to improve cancer treatment outcomes across a number of cancers with common biological/molecular mechanistic pathways. A more complete picture of an individual’s tumor genome may also be integrated within existing frameworks including age, disease burden, and histologic/molecular features for developing more effective cancer treatment and management strategies^4^.

## Published reviews, recommendations and guidelines

This approach to cancer therapy has received widespread interest from patients, healthcare providers and payers^9^. Despite the interest from a wide range of perspectives, there is limited guidance in the form of evidence-based guidelines and recommendations. In order to provide guidance on the best-available evidence of the clinical utility, the Blue Cross and Blue Shield (BCBS) Technology Evaluation Center released in 2013 a review of the implementation of multiple molecular testing in clinical decision making^10^. The available evidence summarized is based on three observational studies reporting outcomes in study participants who received molecularly targeted treatment based on the results of multi-marker tumor panels^11^^-^ 13. The BCBS review also discussed an ongoing randomized controlled trial designed to evaluate the clinical utility of treatment decisions directed by multi-marker tumor panels^14^. None of the commercially available tests (Table 2) were evaluated in any of the studies reviewed in the BCBS report.

In a working paper, UnitedHealth spoke to the larger contextual issues of using molecular testing in clinical oncology. A broad emphasis was placed on the expanding opportunities for molecularly targeted therapy using tumor genome sequencing and for improving the process of determining the effectiveness of molecular testing^15^.

## Analytic validity

The analytic workflow of FoundationOne™, hybrid-capture libraries sequenced with Illumina HiSeq2000, was shown to have an analytic sensitivity between 95% and 99% (depending on the allele frequency) and a positive predictive value greater than 99% using pooled cell lines as the reference standard 16. Using Sanger sequencing as a reference standard, the AsuraGen® SuraSeq™ analytic workflow incorporating PCR-based enrichment followed by NGS with Illumina and Ion Torrent massively parallel sequencing platforms had an analytic sensitivity between 93.8% and 100% and specificity between 95.3% and 100% in 38 FFPE colorectal tumor resections^17^. No information on the validation of post-analytic factors (variant calling) was described on the websites of any tests^18^^-^ 20.

There appears to be consistency in the pre-analytic factors across the commercially available tests related to tissue sample requirements, collection and preservation (Table 2). All the organizations listed in Table 2 report the sequencing is done in Clinical Laboratory Improvement Amendments (CLIA) certified facilities. While this does provide a limited amount of standardization, the fundamental requirements to establish analytic validity requires a reliable reference standard to align and annotate massively parallel sequence data^6^^,^ 7. Proper alignment against the reference genome is critical for integrating NGS in the genomics-driven medicine paradigm. Currently, there are no universally accepted reference standards for genome alignment, and future analyses comparing different alignment approaches are needed to inform the analytic sensitivity and specificity of existing computational approaches that characterize the full spectrum of genomic alterations detected using NGS (e.g. mutations, insertion/deletions, and copy number variants). Analytic validation of the computational procedures applied to additional post-analytic processes (e.g. clinical annotation and interpretation) require the same level of quality-assurance and evidence-based evaluation as the alignment algorithms.

## Clinical validity

Table 3 shows the vast array of molecular targets included in the commercially available tumor panels, with only 34 genes included in all three tests (Figure 1). FoundationOne was reported to detect at least one clinically actionable variant in 76% of samples (N=2200) with an average of 1.57 clinically actionable variants detected per sample (range: 0 to 16)^16^. The frequency at which clinically actionable variants are detected bridges aspects of clinical validity and clinical utility. However, direct evidence of the association of each marker and treatment response is currently lacking for the many possible on- and off-label therapeutic options across all types of cancers. The absence of well-defined clinic effects (treatment response) leaves room for a wide-spectrum of interpretations of the variants identified with potential targeted therapies as well as variants of unknown significance and how gene-gene interaction in both up-stream and down-stream variants may affect response to targeted therapies. Critical to establishing clinical validity is knowledge of the specific clinical effect. Standardized criteria that can be systematically applied during the clinical annotation is necessary for determining if the variant is clinically actionable (i.e. can be matched to an available targeted therapy).

**Common molecular markers included in commercially available test.**
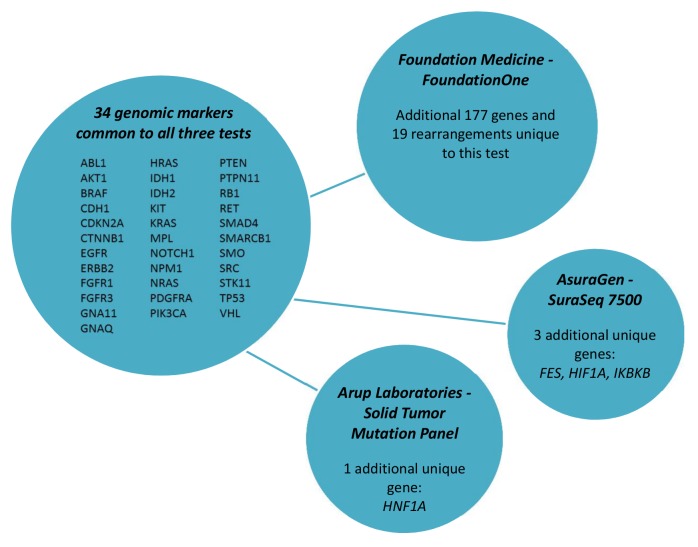

There is a growing body of evidence identifying genomic alterations common across multiple cancer types^2^. Such variants may be associated with various clinical effects (e.g. diagnostic, prognostic, or predictive of treatment response/toxicity) or may have more than one effect. Therefore, evidence is needed to not only determine the presence of potentially actionable variants in multiple cancer types, but more importantly, to determine whether an actionable variant matched with a targeted therapy is associated with the same treatment response regardless of cancer type. This will help standardize the interpretation of test results; ultimately influencing clinical utility. The iterative process of cultivating new markers into established panels as emerging evidence fills in the gaps in the current understanding of the underlying molecular pathways adds additional challenges to systematically evaluating the clinical validity of these tests.

**Figure:**
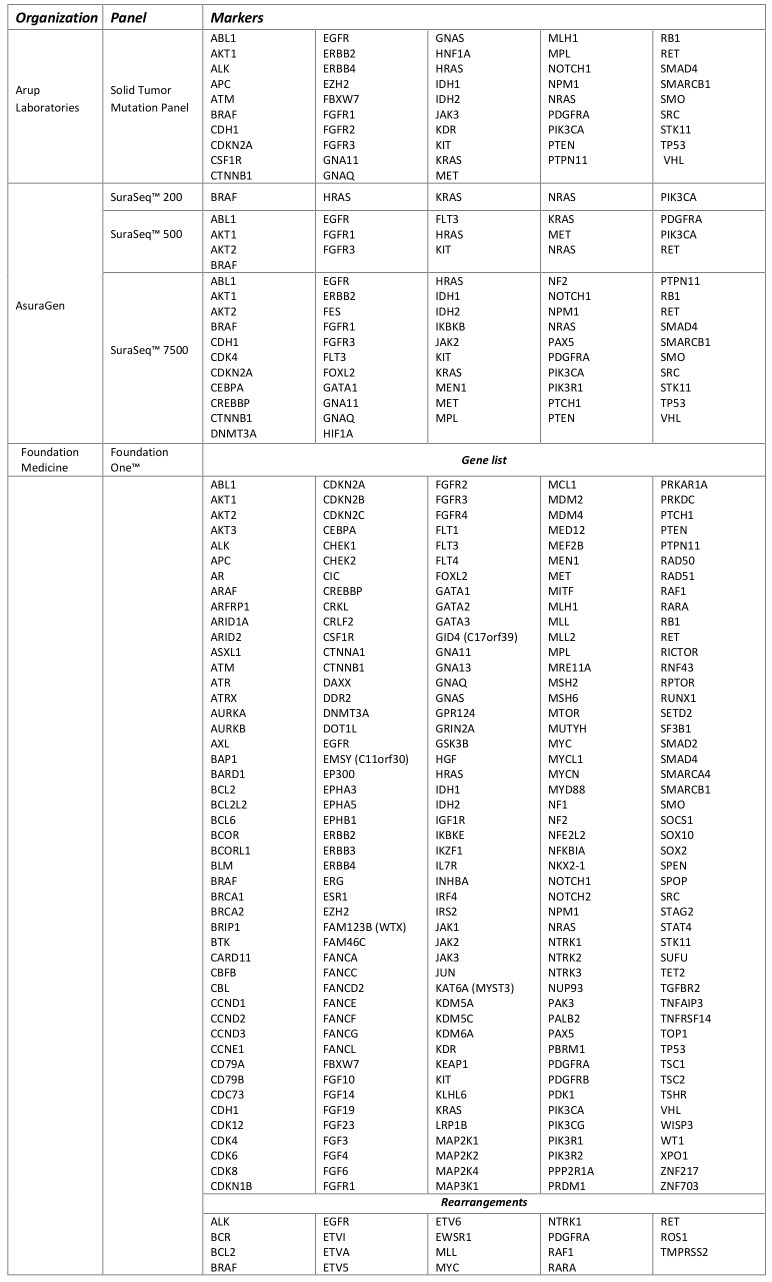
**Table 3. Multi-marker tumor panel composition**

## Clinical utility

**Benefits**

At this time there is no evidence of improved treatment outcomes from using the tests described in Table 2 to direct molecularly targeted therapy in solid-tumors. In addition to the absence of evidence of clinical utility for the commercially available tests, there is also a need to further explore the validity and utility of the underlying hypothesis driving the development and implementation of these tests. One challenge in developing studies investigating the general hypothesis as well as establishing clinical utility is the limited generalizability due to diversity in the evolving panel composition and variation in the clinical interpretation and treatment recommendations (i.e., rule-based or tumor-board based). There is a need to standardize the composition of panels and develop standard criteria for classifying variants as clinically actionable. It may also be necessary to prioritize specific variant/therapy combinations when multiple variants with associated therapies are identified, as there may be overlapping toxicities and drug-drug interactions. Characteristics of studies investigating clinical utility, including molecular heterogeneity of study participants as well as specific study designs will also impact generalizability.

**Harms**

None of the test descriptions included details of patient consent or how incidental findings would be communicated to clinicians or patients. As these tests are intended to be implemented at the point of care, such protocols may be left to the ordering physician and their home institution to determine processes for informed consent, delivery of appropriate pre-test genetic counseling, and disclosure of incidental findings. Given the analytic workflow of the available tests (Table 2) and diverse panel composition (Table 3) it is uncertain how the ACMG recommendations^21^ for reporting incidental findings from germline sequencing apply to tumor sequencing. This is not to say tumor DNA sequencing is immune to potential incidental findings. There remains the possibility of detecting significant germline variants through subtractive analyses comparing sequenced normal tissue with tumor sequences^22^. This would require both tumor tissue and normal tissue to be sequenced which is not currently described by the companies offering the commercial tests.

Another potential harm from implementing this testing and treatment strategy arises from uncertainty in the cost-benefit ratio of the off-label use of expensive chemotherapeutic drugs. As mentioned previously, these tests are currently being offered to patients with advanced disease in whom time spent delivering ineffective therapy may pose a significant risk as a wasted opportunity. Many of the available drugs are associated with well characterized adverse effects (e.g. cardiotoxicity from the kinase inhibitors)^23^^,^ 24. When the possibility of these adverse effects are considered alongside the unknown effectiveness of the off-label use of these drugs in various tumor types, there is the real potential of clinical and economic harms.

## Ongoing studies

Several ongoing clinical trials have incorporated the commercially available tests (Table 4). Designed as feasibility assessments, two non-randomized trials are using FoundationOne (Foundation Medicine) to identify participants with metastatic breast cancer (IMAGE)^25^ and participants with various solid tumor types^26^ that could benefit from molecularly targeted therapy. The objectives for the IMAGE trial is to determine the time to report molecular profiling results, the ability to make treatment suggestions based on the molecular profile and why clinicians/patients followed treatment recommendations from a molecular profiling tumor board. The primary objectives of the second study^26^ is to assess the number of participants screened, number of tests attempted and number of successful tests, and the ability or inability to implement NGS results-based non-FDA-approved treatment plan. Foundation Medicine is also participating in another non-randomized trial evaluating the performance of molecularly targeted therapy based on NGS results compared to prior therapy (WINTHER)^27^. A unique feature of the WINTHER trial is the “N of 1” design in which progression-free survival in participants is compared between periods when they received targeted therapy versus periods prior to their entry in the study.

**Figure:**
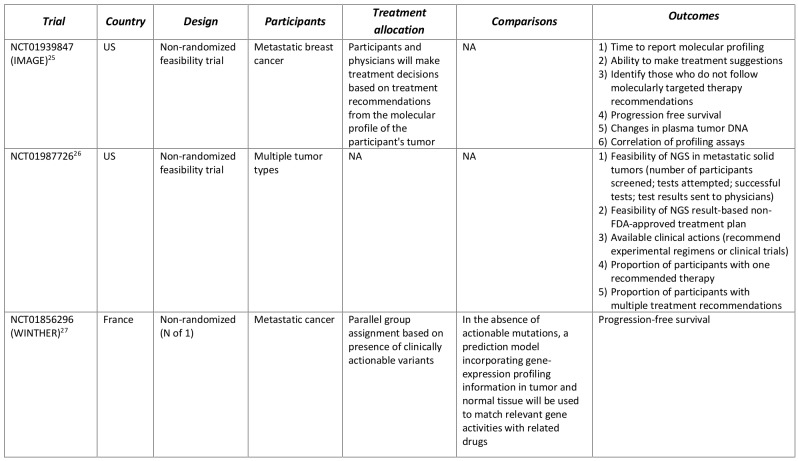
**Table 4. Clinical trials incorporating commercially available multi-marker tumor panels for making treatment decisions.**

## Conclusions

Recent advances in sequencing technology provide new opportunities for genomic medicine. With these opportunities come the promises of personalized medicine that are changing oncology practice. However, aspects of analytic and clinical validity and clinical utility of the current paradigm shift has yet to be clearly established. Developing the necessary evidence to establish clinical validity and utility may require novel thinking to adapt to the dynamic challenges associated with implementing NGS tumor sequencing into clinical practice. Researchers must also address several limitations in the underlying concepts of this approach including patient selection, analytic workflows, characteristics of the tumor panels (performance characteristics and panel composition), study design, and outcome selection. Several ongoing clinical trials are investing both the feasibility and utility of incorporating NGS technology into clinical practice and will help define the evidentiary standards for evaluating such tests.

## Methods

The GAPP Knowledge Base (GAPP KB) was searched using the term “next-generation sequencing” to identify commercially available multi-marker solid tumor tests using NGS technology. A Google search supplemented the GAPP KB search for eligible tests as well. The following search string was used to search PubMed to identify relevant systematic reviews and guidelines:

(((neoplasms/therapy[mesh]) OR (neoplasms/genetics[mesh]) OR (neoplasms/diagnosis[mesh])) AND ((tumor markers, biological/genetics[mesh]) OR (molecular targeted therapy/methods[mesh]) OR (genetic testing[mesh]) OR (DNA mutational analysis[mesh]))) AND (humans[mesh])

In addition to searching PubMed, the commercially available tests websites were searched to identify relevant literature pertaining to the analytic validity, clinical validity, and clinical utility of the respected test. Ongoing studies were identified in clinicaltrials.gov by searching on the trade name of each commercially available test and company offering the tests described in Table 2. This was supplemented by using the term “next-generation sequencing” to identify additional studies not using any of the tests listed in Table 2.
